# Overexpression of a *GmCnx1* Gene Enhanced Activity of Nitrate Reductase and Aldehyde Oxidase, and Boosted Mosaic Virus Resistance in Soybean

**DOI:** 10.1371/journal.pone.0124273

**Published:** 2015-04-17

**Authors:** Zheng Zhou, Hongli He, Luping Ma, Xiaoqian Yu, Qian Mi, Jingsong Pang, Guixiang Tang, Bao Liu

**Affiliations:** 1 Institute of Crop Science, College of Agriculture and Biotechnology, Zhejiang University, Hangzhou, China; 2 Molecular Epigenetices of the Ministry of Education (MOE), Northeast Normal University, Changchun, China; Murdoch University, AUSTRALIA

## Abstract

Molybdenum cofactor (Moco) is required for the activities of Moco-dependant enzymes. Cofactor for nitrate reductase and xanthine dehydrogenase (*Cnx1*) is known to be involved in the biosynthesis of Moco in plants. In this work, a soybean (*Glycine max *L.) *Cnx1 *gene (*GmCnx1*) was transferred into soybean using *Agrobacterium tumefaciens*-mediated transformation method. Twenty seven positive transgenic soybean plants were identified by coating leaves with phosphinothricin, bar protein quick dip stick and PCR analysis. Moreover, Southern blot analysis was carried out to confirm the insertion of *GmCnx1* gene. Furthermore, expression of *GmCnx1* gene in leaf and root of all transgenic lines increased 1.04-2.12 and 1.55-3.89 folds, respectively, as compared to wild type with *GmCnx1* gene and in line 10 , 22 showing the highest expression. The activities of Moco-related enzymes *viz *nitrate reductase (NR) and aldehydeoxidase (AO) of T_1_ generation plants revealed that the best line among the *GmCnx1* transgenic plants accumulated 4.25 μg g^-1^ h^-1^ and30 pmol L^-1^, respectively (approximately 2.6-fold and 3.9-fold higher than non-transgenic control plants).In addition, overexpression of*GmCnx1*boosted the resistance to various strains of soybean mosaic virus (SMV). DAS-ELISA analysis further revealed that infection rate of *GmCnx1* transgenic plants were generally lower than those of non-transgenic plants among two different virus strains tested. Taken together, this study showed that overexpression of a *GmCnx1* gene enhanced NR and AO activities and SMV resistance, suggesting its important role in soybean genetic improvement.

## Introduction

Molybdenum (Mo) forms a complex with the molybdopterin compound, which consequently forms a cofactor named molybdenum cofactor (Moco). All Mo-containing enzymes characterized to this end, besides nitrogenase contain a pterin-type cofactor[1]. In plants, six gene products have been identified to be involved in the Moco biosynthesis, which can be divided into four steps. The basic step of Moco biosynthesis begins with the conversion of guanosine triphosphate (GTP) to cyclic pyranopterin monophosphate (cPMP, previously identified as precursor Z) with the catalysis of *Cnx2* and *Cnx3* in the mitochondria, while all subsequent steps proceed in the cytosol. In the next step sulfur is transferred to cPMP so as propagate the intermediate MPT in a reaction catalyzed by copper (Cu) and the enzyme MPT synthase, a complex consisting of *Cnx6* and *Cnx7* subunits. The third step involves the insertion of Mo into MPT-AMP with adenylation of molybdopterin. In the final step, a product-substrate channel is built by the transference of MPT-AMP to the N-terminal domain of *Cnx1* (Cnx1-E). Cnx1-E then cleaves the adenylate, releasing Cu and inserts Mo, thus producing active Moco [2,3].

Molybdenum containing enzymes play vital role in nitrogen assimilation, synthesis of phytohormones, detoxification and purine metabolism[4]. Although more than fifty Mo-containing enzymes are known, only the functions of five Mo-enzymes have been exploited so far, which include: nitrate reductase (NR;EC 1.7.1.1), aldehyde oxidase (AO; EC 1.2.3.1), xanthine dehydrogenase (XDH; EC 1.17.1.4), sulphite oxidase (SO; EC 1.8.3.1), and mitochondrial amidoxime reductase (mARC) [4]. Among these five Mo-enzymes, NR and AO are considered as vital enzymes involved in various processes of plant growth and regulation.

NR is a 200 kDa cytoplasmic enzyme, and consists of three functional domains: the N-terminal domain associated with Moco, the central haem-binding cytochrome *b*_*5*_ domain, and the C-terminal FAD-binding domain. This dimerization is Moco dependent [5]. The three domains form three redox centers catalyzing the transfer of electrons from the reduced NAD(P)H via FAD, haem and Moco to nitrate.NR catalyses the first step in nitrate assimilation, a pathway that is of key importance for plant nutrition [6,7].The regulation of NR involves both transcriptional and post-translational mechanisms regulating the amount as well as activity of NR protein.

AO is a cytoplasmic enzyme with an apparent molecular mass of 300 kDa. The FAD, Fe, and Moco acts as aprosthetic groups and bind to the enzyme with the propertion of 4:1:1 [8].AO isoform (AAO3) acts on abscisic aldehyde, which is the native precursor of the plant hormone abscisic acid. Abscisic acid is believed to be involved in many developmental processes as well as for a variety of abiotic and biotic stress responses [9–11]. Due to broad substrate specificity AOs are involved in additional metabolic processes other than phytohormone synthesis. Detoxification reactions and pathogen response may be good candidates for these additional functions [12]. Recently it was shown that plant AOs produce H_2_O_2_ in response to drought stress and ABA treatment [13]. Hence, AO enzymes in plants are essential for many physiological processes.

However, recent studies about the physiological and biochemical functions of *GmCnx1* Moco biosynthesis genes have been minute. Previous literatures have shown the importance of *Cnx1* in forming a stable Moco, and that *Cnx1* binds to actin filaments of plants cytoskeleton located on the membrane with N-terminal domains [14]. In addition, expression of a senescence-associated gene *Ntdin* was related to synthesis of Moco in tobacco, resulting in the increase of the content of Moco. NR and XDH activity was also improved in transgenic *Ntdin* plants [15]. It was also found that expression of the Mocosulfide enzyme in soybean enhanced drought resistance, which in turn improved its yield [16].Furthermore, overexpression of *Cnx1* gene was related to SMV infection based on whole-genome microarray analysis [17].

Although studies regarding the function of *Cnx1* in Moco biosynthesis have been widely conducted, its specific functions in enhancing NR, AO activities and SMV resistance have not been explored yet. Therefore, the main aim of this study was to elucidate the potential functions of *GmCnx1* in enhancing NR, AO activities and SMV resistance using the *Agrobacterium tumefaciens*-mediated transformation, and characterization of the *GmCnx1*-overexpressing transgenic soybean plants.

## Materials and Methods

### Plant materials and growth

Soybean cultivar, Tianlong 1, was used as the recipient for genetic modification. The *Agrobacterium tumefaciens*-mediated transformation in soybean was carried out according to Zhang et al.[18] and Paz et al.[19] to get T_0_ seeds, and the T_1_ seeds were harvested from self-pollination of T_0_ progeny.

The wild type (WT) and transgenic*GmCnx1* gene soybean plants were grown in a controlled greenhouse at Zijingang campus experimental farm in Zhejiang University, PR China, in 2013. The growth temperature of soybean was 28/20 ± 1°C (day/night) with a 16 h photoperiod under fluorescent white light. Plants were daily supplied with half-length Hoagland’s nutrient solution [20] during growth.

### Isolation of the cDNA sequence and the construct of *GmCnx1* binary vector

A gene-specific primer pair (5’-GGGGACAAGTTTGTACAAAAAAGCAGGCTTCACC-3’ and 5’-GGGGACCACTTTGTACAAGAAAGCTGGGTG-3’), was designed according to the predicted sequence of *Cnx1* (NM_001255600; NCBI Reference Sequence). About 5 μg total RNA in leaf was reversely transcribed into first-strand cDNA by ThermoScripy Kit (Invitrogen, USA). The PCR product was sequenced and inserted into pDONOR221 (Invitrogen) and then transferred to a pB7FWG2 vector via LR recombination reaction of the Gateway system.

### Identification of putative transgenic plants

Non-transgenic soybean plants and *GmCnx1* transgenic soybean plants both produced the amplified band of *GmCnx1* gene by PCR analysis, since the target gene is endogenous. The *bar* gene and *GmCnx1* gene which were contained between LB and RB on the vector, were transferred together into soybean plants, so we selected *bar* gene as the identification standard for transgenic plants. The following methods were applied:

Firstly, about 10 d after the plantlets were transferred to soil, the T_1_ plants were screened for tolerance to the herbicide *Basta* by application of a 135 mg/L solution with a cotton swab lightening the upper surface of the half euphylls and labeling the other half with pen. Leaf tissue was recorded for herbicide tolerance at 6 or 7 d post lightening.

Secondly, quick detection of the bar protein was carried out by using Quickstix Kit for *bar* gene according to the manufacturer’s instructions (EnviroLogix Inc., USA). The presence of a test line (second line) on the membrane strip between the control line (common to all, including the non-transformed control) and the protective tape indicated the expression of the foreign bar protein in the transgenic plants.

Thirdly, bar-resistant transgenic plants were first identified by PCR amplification [21] with primers for the *bar* gene. Primers were designed according to the nucleotide sequence of selective maker gene *bar* (5’-CAGCTGCCAGAAACCCACGT-3’ and 5’-CTGCACCATCGTCAACCACT-3’). Amplification of gene *bar* used 2×Taq Master Mix (YEASEN, Shanghai, China). After completion of PCR amplification, the amplified product was detected on 1.5% agarose electrophoresis.

### Validation of transgenic soybean plants by Southern blot analysis

Genomic DNA was extracted from leaf of non-transgenic and the putative T_1_ *GmCnx1* transgenic plants (based on herbicide resistance, mentioned above) by the method described by Edwards et al.[22] with minor modifications. Plasmid DNA and genomic DNA were digested with restriction enzymes *EcoR* I and *Hind* Ⅲ, respectively, at 37°C overnight. Digested DNA was separated on 0.8% (w/v) agarose gel and blotted onto Hybond N^+^ nylon membrane. The membrane was hybridized with a DIG-labelled *GmCnx1*-specific probe (5’-CGAGTATCCCGTCGTTG-3’ and 5’-TTCCAGGTAGCCCAAAA-3’) at 68°C for 16 h. The hybridized membrane was washed and detected according to the protocol of High Prime DNA Labeling and Detection Starter Kit I (Roche, USA).

### qRT-PCR analysis of the transgenic soybean plants

Fresh tissues of two-week-old T_1_transgenic and non-transgenic soybean plants were collected in liquid nitrogen before isolation of RNA. Total RNA was isolated leaf and root using Trizol reagent (TaKaRa, Dalian, China). First strand cDNA was synthesized with 1 μg of purified total RNA using PrimeScript RT reagent Kit with gDNA Eraser (TaKaRa). Real-time PCR reaction was carried out according to SYBR *Premix Ex Taq*II (TaKaRa). The gene-specific primers of *GmCnx1* (5’-CCTGGAAACCCTAATGCTGT-3’ and 5’-CACATCTGCAGGAACTGCTT-3’) were used in the RT-PCR and gene—specific primers of *actin11* (5’-ATCTTGACTGAGCGTGGTTATTCC-3’ and 5’-GCTGGTCCTGGCTGTCTCC-3’) were amplified as internal control to quantify the relative amounts of cDNA [23]. The amplifications were performed on CFX96 Real Time System (Bio-Rad, Hercules, CA, USA) and the relative expression was calculated by 2^-ΔΔCt^ method [24]. Significant differences of all data were analyzed using DPS software.

### NR activity assay

NR activity was measured according to the method described previously[25]. The NR activity of plant crude extracts was assayed using leaf of non-transgenic and T_1_ *GmCnx1* transgenic plants, which were homogenized in extraction buffer and centrifuged for 15 min at 4,000 rpm. The supernatants were collected and added to the reaction buffer. After incubation at 25°C for 30 min, the reaction was stopped by 1 ml 1% sulphanilamide. The mixture was centrifuged for 5 min at 5,000 rpm and the supernatant was used to detect nitrite production by reading the absorbance at 540 nm after addition of *N*-(1-naphthyl) ethylenediamine dihydrochloride. NO_2_^-^ was quantified to calculate NR activity.

### AO activity assay

Detection of AO activity was performed by using Plant Aldehyde Oxidase (AO) Elisa Kit (TSZ Co. Ltd., USA) according to the manufacturer’s instructions. Enzyme linked immunosorbent assay——sandwich technique was employed in the experiments. Briefly, standard wells and testing sample wells were set. All of 50μl standards, 50μl samples and 100μl HRP-conjugate reagents were added into standard wells and sample wells, respectively. Cover with an adhesive strip and incubate for 60 min at 37°C. Aspirate each well and wash, repeating the process five times. Wash by filling each well with Wash Solution (400μl) using a squirt bottle. Add 50μl chromogen solution ‘A’ and 50μl chromogen solution ‘B’ to each well. Gently mix and incubate for 15 min at 37°C. Add 50μl Stop Solution to each well. The color in the wells should change from blue to yellow. Read the OD_450_ using spectrophotometer within 15 min. The concentration of AO in the samples is determined by comparing the OD_450_ of the samples to the standard curve.

### Resistance identification and DAS-ELISA of SMV

The friction inoculation method was employed[26]. Leaves of SMV-1 and SMV-7 strains were contributed by Professor Bao Liu of Northeast Normal University. Soybean plants were grown in soil pots for 20 d prior to inoculation. Inoculums were prepared by grinding infected soybean leaf tissues with emery in 0.01 mol L^-1^ phosphate buffer, pH 7.0. The unrolled unifoliate leaves were inoculated by gently rubbing with the inoculums. Five soybean plants were infected with two different SMV strains for each plant line and three biological replicates were used for inoculation and sampling. Symptoms of SMV were observed after three weeks. ELISA assay was performed, which was referred to as Soybean Mosaic Virus (SMV)-DAS ELISA (AC Diagnostics, Inc., USA). The OD_405_ of T_1_ *GmCnx1* transgenic plants (P) and non-transgenic plants (N) were analyzed, and if the ratio of the sample and negative control (P/N) was higher than 2, the sample plant was rendered as susceptible, while if the ratio was lower than 2 it was considered as resistant.

## Results

### Phylogenetic analysis and homologous sequence alignment of the *GmCnx1* genes

**Fig 1:**
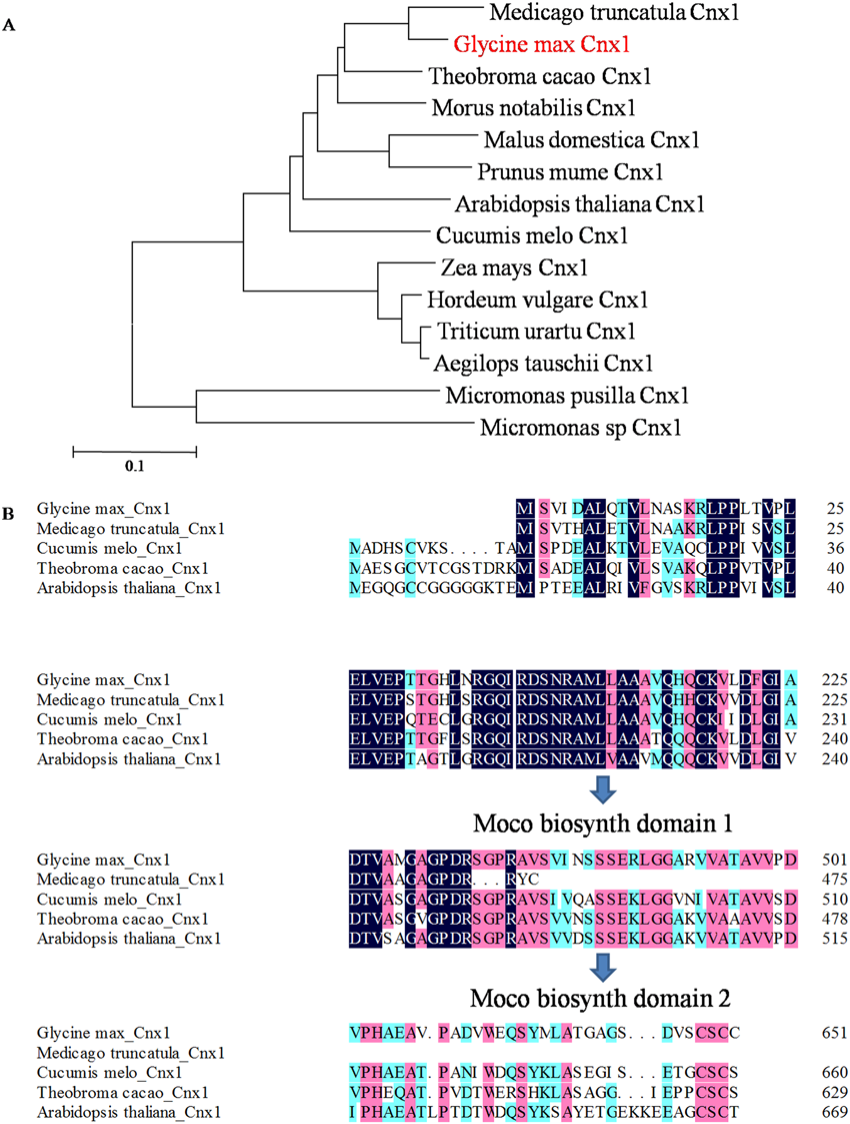
Phylogenetic tree of *GmCnx1* gene and alignment of amino acid residues from different species. (A) Phylogenetic tree constructed by multiple sequence alignments of the *Glycine max Cnx1* and those of other *Cnx1* proteins. The sequences used are: *Medicago truncatula Cnx1*, *Theobroma cacao Cnx1*, *Morus notabilis Cnx1*, *Malus domestica Cnx1*, *Prunus mume Cnx1*, *Arabidopsis thaliana Cnx1*,*Cucumis melo Cnx1*,*Hordeum vulgare Cnx1*, *Zea mays Cnx1*, *Triticum urartu Cnx1*, *Aegilops tauschii Cnx1*, *Micromonas sp Cnx1* and *Micromonas pusilla Cnx1*(Accession nos AET00019, EOY33170, EXB53803, XP_008385638, XP_008231326, EEH56197, XP_008451034, AAF73075, ABB30174, EMS55337, EMT03164, ACO61591 and AED92917.1, respectively). (B) Through the analysis of two Moco domains and alignment of amino acid residues, encoding protein sequences of *GmCnx1*, *Medicago truncatula Cnx1*, *Cucumis melo Cnx1*, *Theobroma cacao Cnx1* and *Arabidopsis thaliana Cnx1* had high sequence similarity.

The phylogenetic tree **(** Fig 1a) was constructed by Mega software version 5.0 using the neighbor-joining method [27].As expected, *Glycine maxCnx1* and *Medicago truncatulaCnx1*both belonging to Legume, *Papillionoideae*, had the closest genetic relationship. Two Moco domains in protein coded by *Glycine max Cnx1* gene were found by online SMART software of EMBL (http://smart.embl-heidelberg.de/). The locations of the two domains were confirmed by DNAMAN software. A comparison of amino acid sequences **(** Fig 1b) revealed that *Glycine max Cnx1* shared high sequence similarity with the *Cnx1* gene of *Medicago truncatula*, *Cucumis melo*, *Theobroma cacao* and *Arabidopsis thaliana*. The sequence similarity of the proteins coded by the *Cnx1* genes was higher than 60% and amino acid sequences at the*Cnx1* conserved domains in particular were highly conserved.

### Genetic transformation and identification of transgenic soybean plants

**Fig 2:**
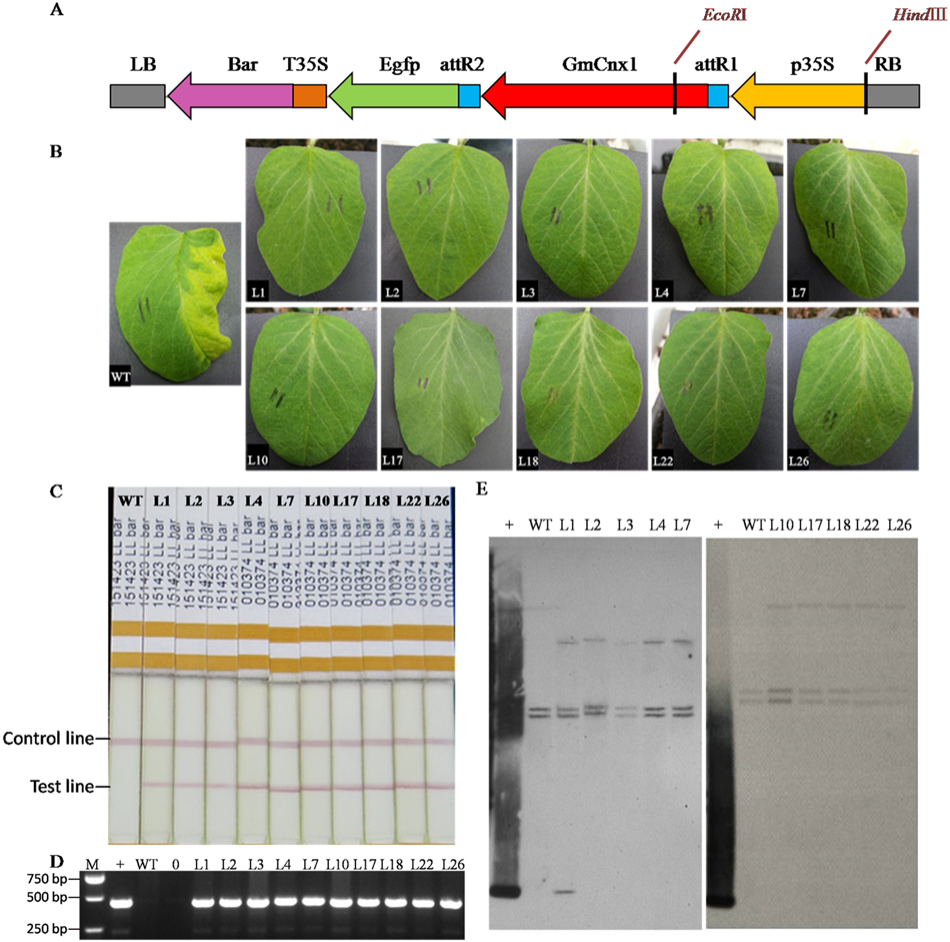
Recombination expression vector of pB7FWG2-*GmCnx1* and determination of *GmCnx1* transgenic soybean plants. (A) Vector contained selective maker gene (*bar*) coded phosphinothricin acetyltransferase (PAT) and showed resistance to the herbicide phosphinothricin, green fluorescent protein gene (*Egfp*) and *GmCnx1* gene. LB, left border; RB, right border; p35s, promoter; T35S, terminator. (B) T_1_ transgenic lines were confirmed by coating leaves with Phosphinothricin. (C)T_1_ transgenic lines were confirmed by bar protein quick dip strip. (D) T_1_ transgenic lines were confirmed by PCR. (E) Southern blot analysis of transgene copy number in T_1_ transgenic soybean and WT. Genomic DNA and plasmid DNA was digested with restriction enzyme *EcoR* Iand *Hind*Ⅲ. The probe was used for *GmCnx1*. M, DL2000 marker; +, positive control (plasmid DNA); WT, negative control, non-transgenic plants; 0, blank. L1, L2, L3, L4, L7, L10, L17, L18, L22 and L26 represent the *GmCnx1* transgenic line numbers.

The pB7FWG2-*GmCnx1* vector (Fig 2a) was constructed and introduced into soybean via *Agrobacterium tumefaciens*-mediated transformation (S1 Fig). A total of twenty seven independent putative soybean transgenic lines were generated. Ten positive lines 1, 2, 3, 4, 7, 10, 17, 18, 22 and 26 which had sufficient seeds were selected for subsequent experiments.T_1_putative transformants were identified by coating leaves with Phosphinothricin (Fig 2b),bar protein quick dip strip (Fig 2c) and PCR (Fig 2d). Southern blot (Fig 2e) of T_1_ generation revealed successful integration of the target gene into the soybean genome, which showed stable overexpression of *GmCnx1*. Importantly, the transgene construct was a single copy in line 2, 3, 4 and 7.

### Expression of *GmCnx1*in transgenic soybean lines

**Fig 3:**
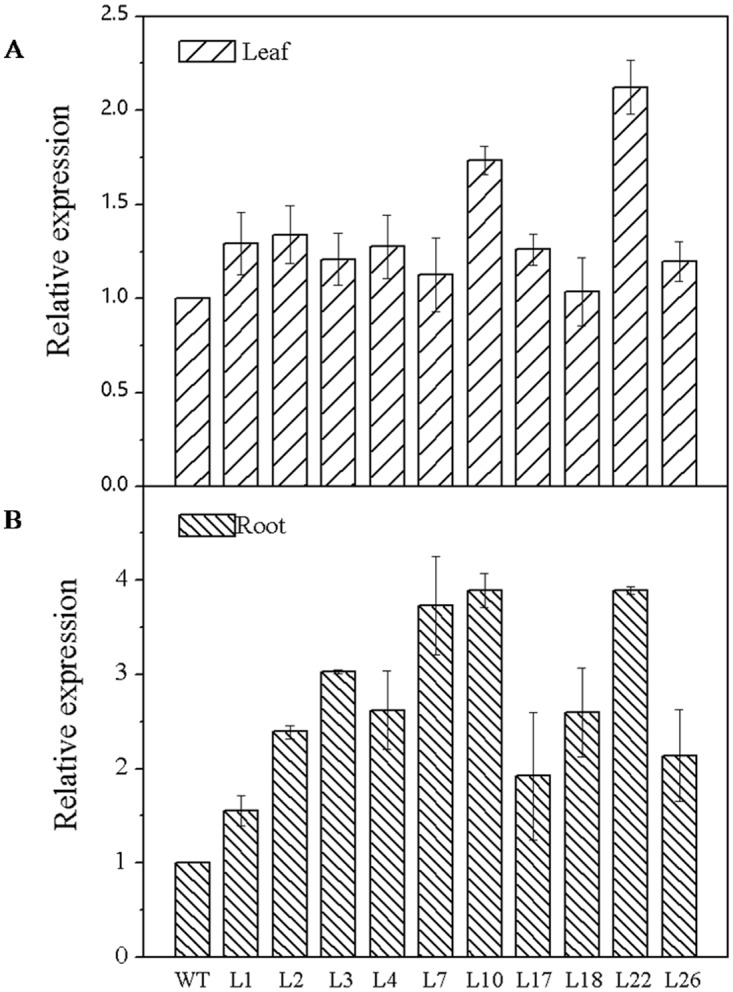
Expression of target gene *GmCnx1* in all two-week-old leaf (upper) and root (bottom) of WT and T1*GmCnx1*transgenic soybean plants. WT, negative control, non-transgenic plants. L1, L2, L3, L4, L7, L10, L17, L18, L22 and L26 represent the *GmCnx1* transgenic line numbers. Values are the mean ± SD (n = 3). Error bars represent standard errors of means from three replicates.

The expression of *GmCnx1* was investigated by qRT-PCR in leaf and root under normal growth conditions. The results (Fig 3) showed that the expression of *GmCnx1* gene increased from 1.04 to 2.12 fold in leaf and 1.55 to 3.89 fold in root of all transgenic positive plants in comparison to the WT, with line 10 and 22 exhibiting the highest expression levels among the different transgenic lines. These results indicated that the*GmCnx1* gene expression was more significantly upregulated in root than in leaf.

### NR and AO activity in the *GmCnx1*-overexpressing transgenic soybean lines

**Fig 4:**
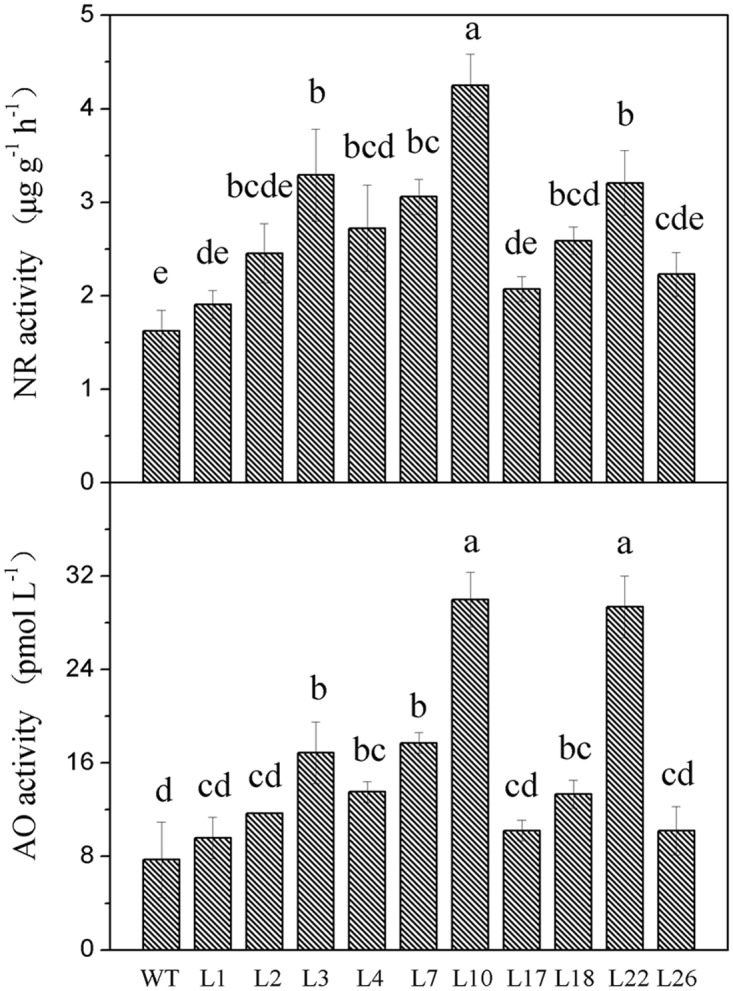
NR (upper) and AO (bottom) activities in leaf of both WT and T1 *GmCnx1*transgenic soybean plants. Data were calculated by significance analysis method, and the significant differences were compared with the control. WT, negative control, non-transgenic plants. L1, L2, L3, L4, L7, L10, L17, L18, L22 and L26 represent the *GmCnx1* transgenic line numbers. Values are the mean ± SD (n = 3). Error bars represent standard errors of means from three replicates. The same letter at each bar means no significant difference at *P*< 0.05.

As NR and AO activity was causally linked with the biosynthesis of Moco, they were measured in leaves of WT and T_1_ transgenic plants. The results showed that, NR and AO activities increased in *GmCnx1* transgenic plants compared with WT (Fig 4). In particular, for NR activity, the *GmCnx1* transgenic soybean plants produced high level (4.25 μg g^-1^ h^-1^) in line 10, which were 2.6-fold higher than the non-transgenic plants (1.62 μg g^-1^ h^-1^), and for AO activity, the level in the leaf was 3.9-fold higher in *GmCnx1* transgenic soybean plants (30.00 pmol L^-1^) in line 10 compared with the non-transgenic plants (7.71 pmol L^-1^). That was consistent with the expression of *GmCnx1* gene in leaf and root. Taken together, it can be concluded that the different*GmCnx1* levels among the different transgenic lines correspond to similar differences at NR and AO activities in leaf.

### Resistance to SMV was increased in *GmCnx1* transgenic soybeanlines

**Fig 5:**
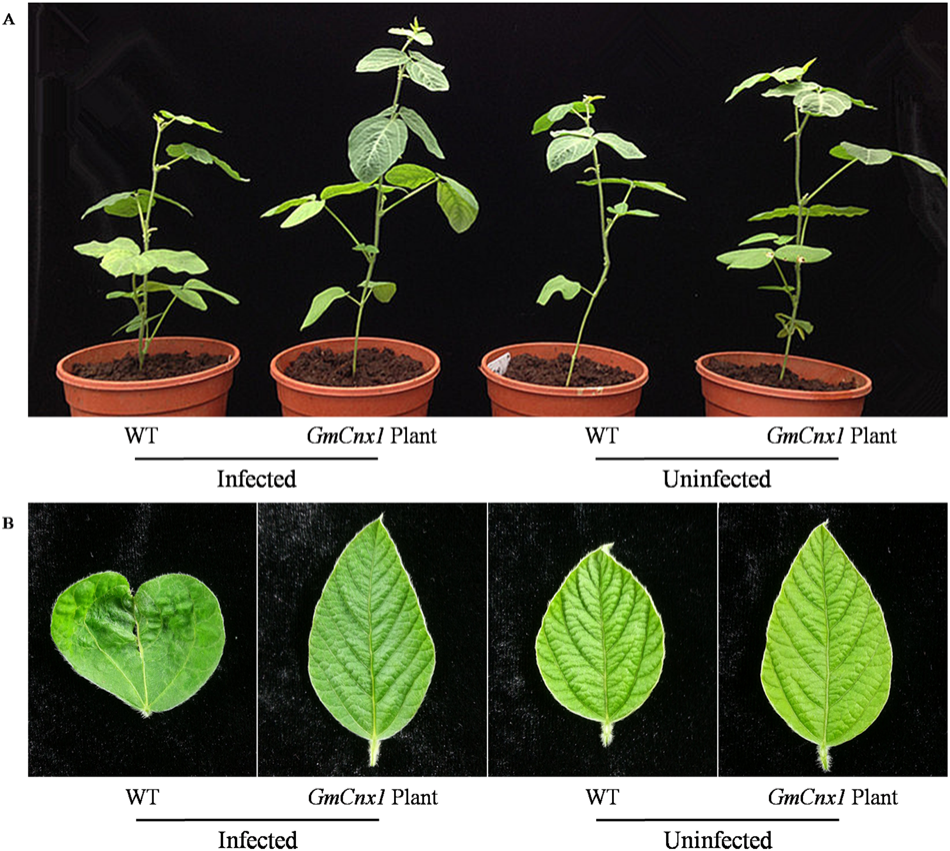
Comparison of SMV resistance in soybean three weeks after friction inoculation. (A) Growth performance of WT and T_1_ *GmCnx1* transgenic plants before and after infection. (B) Symptoms on tender leaves (top second leaf) of WT and T_1_ *GmCnx1* transgenic plants before and after infection. WT, negative control, non-transgenic plants.

**Fig 6:**
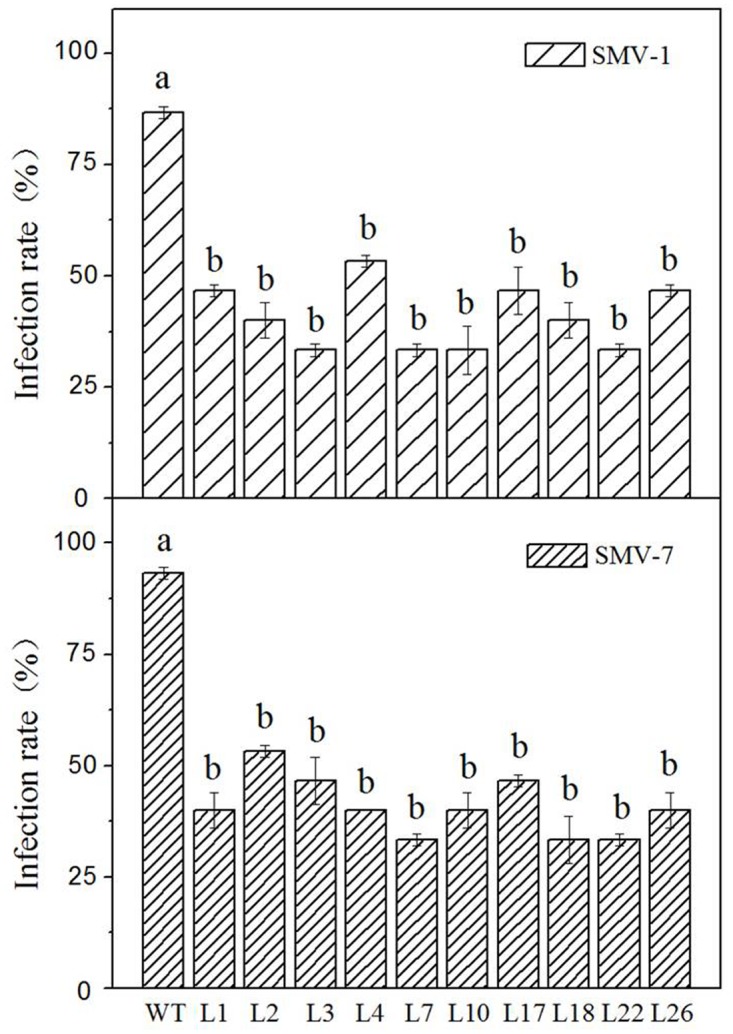
The infection rate of SMV-1 (upper) and SMV-7 (bottom) in both non-transgenic and *GmCnx1* transgenic soybean plants by DAS-ELISA. WT, negative control, non-transgenic plants. L1, L2, L3, L4, L7, L10, L17, L18, L22 and L26 represent the *GmCnx1* transgenic line numbers. Values are the mean ± SD (n = 3). Error bars represent standard errors of means from three replicates. The same letter at each bar means no significant difference at *P*< 0.05.

After being infected by SMV-1 and SMV-7 mosaic virus strains, non-transgenic soybean plants showed dwarfing (Fig 5a), mosaic leaves, browning veins and crimple leaves (Fig 5b). In contrast, leaves of T_1_ transgenic soybean plants remained green, showing no mosaic, shrinkage leaves and other symptoms. Resistance analysis by DAS-ELISA (Fig 6) showed that infection rate of *GmCnx1* transgenic plants were generally lower than those of non-transgenic plants. Specifically, for SMV-1, the infection rates of non-transgenic and10 lines of *GmCnx1* transgenic plants were 87%, 47%, 40%, 33%, 53%, 33%, 33%, 47%, 40%, 33% and 47%, respectively. For SMV-7, the infection rates of non-transgenic and 10 lines of *GmCnx1* transgenic plants were 93%, 40%, 53%, 47%, 40%, 33%, 40%, 47%, 33%, 33% and 40%, respectively. The results above indicated that the *GmCnx1* transgenic soybean lines we generated showed higher degrees of resistance to two SMV strains tested.

## Discussion

Although soybean is one of the most important food and feed crops worldwide, its transformation efficiency is relatively low. In comparison with other crops, there are still several problems that need to be tackled such as difficulties in the regeneration potential of the tissues, low reproducibility, and greater genotypic differences[28].In the light of these issues, we optimized several physical and chemical parameters of the standard *Agrobacterium tumefaciens*-mediated transformation protocol for soybean, and obtained significantly improved results [29].Here, in soybean, we successfully generated transgenic soybean plants for the *GmCnx1* gene, validated the previous function of the gene in enhancing NR activity and unveiled the novel function of the gene in enhancing AO activity and SMV resistance.

Cnx1 is a functional protein with two domains, which are essential for catalyzing the insertion of Mo into MPT in plants [30]. E and G domains that were detected in Arabidopsis were involved in the activity of Cnx1 [14]. In the present study, the comparison of amino acid sequences of Cnx1 between Arabidopsis and soybean shows two highly conserved amino acid motifs involved in biosynthesis of molybdopterin cofactor present in soybean (Fig 1b). Moreover, these two domains were suggested in binding with molybdopterin and form an alpha/beta structure. So it was predicted that these two domains would interact with each other for the function of GmCnx1, thus catalyzing the incorporation of Mo into MPT in soybean.

According to Mendel et al. [31], the third step in the biosynthesis of Moco is catalyzed by the two domain-containing proteins Cnx1 (Cnx1-G, Cnx1-E). Mature Moco can bind to NR and AO. Moco-deficient plant mutants showed a pleiotropic loss of all four Mo-enzymes activities NR, AO, XDH and SO. Moco-mutants have been described in numerous higher plants, for example, in tobacco [32, 33], *Nicotiana plumbaginigolia* [34] and barley [35].*Cnx1* expression was found in all organs of Arabidopsis plants, and it was noted that transgenic *Cnx1*gene could increase activity of NR and content of MPT [14]. Moreover, blockage of Moco biosynthesis due to mutations led to the impairment of essential metabolic functions because of the loss of functions of all Mo-dependent enzymes, which ultimately led to death of the organism [36]. On the other hand, there is paucity of literature regarding AO activities in plants. Early reports showed in potato the presence of two isoenzymes for AO that are able to use diverse aldehydes but not xanthine as substrates [37]. The reduction of ABA levels due to the lack of AO activity had a direct impact on Moco-sulfurase deficiency [38,39]. A barley *cnx* mutant showed a thermo sensitive, wilty phenotype, which was attributed to an ABA deficiency due to the absence of activity of a Moco-dependent AO[40].We found that overexpression of *GmCnx1* gene indeed enhanced activities of NR and AO in the transgenic soybean plants (Figs 3 and 4), consistent with previous studies in other plants. In addition, for evaluation of agronomic traits, three transgenic and three non-transgenic soybean plants were randomly sampled before flowering stage from the experimental greenhouse. Plant height, average leaf length, average leaf width, nodules number, fresh weight of nodules, root and shoot fresh weight, root and shoot dry weight and total biomass were measured (S1 Table).

**Fig 7:**
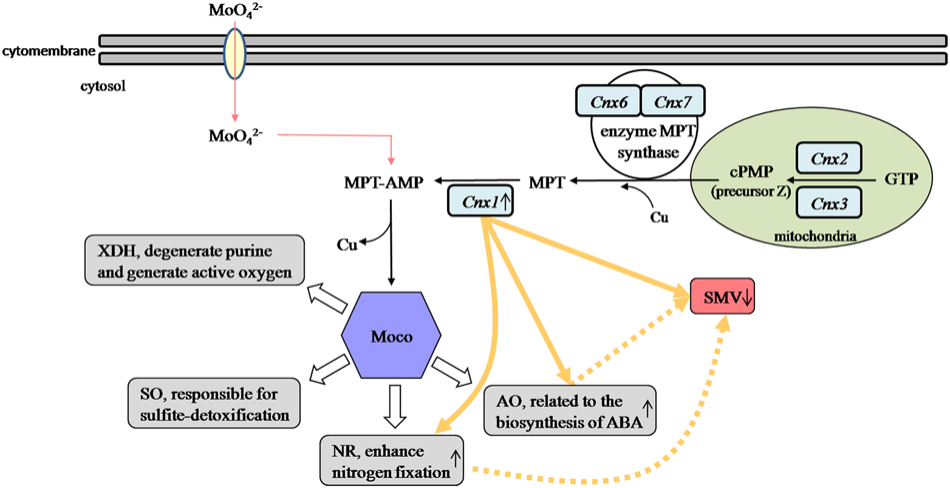
Showing the relationship among *GmCnx1* gene, molybdenum enzymes and SMV. *Cnx1*, *Cnx2*, *Cnx3*, *Cnx6* and *Cnx7* are involved in Moco biosynthesis. Solid arrows represent functions of *GmCnx1* observed in this present work while dotted arrows represent future research prospective and open arrows show Moco-dependent enzymes that have been published in other research work [2,3].

SMV seriously affects yield and quality of soybean globally. Due to its wide distribution, great harmfulness, and difficulties in chemical control, cultivating and planting resistant varieties is an economical, safe and effective way to prevent SMV. In the present study we aimed at checking the relationship between *GmCnx1* gene and its function in plants with regards to SMV resistance. Based on a microarray analysis of gene expression differences between two contrasting genotypes (SMV resistant and SMV susceptible) upon SMV inoculation, *GmCnx1* was selected as a candidate SMV resistant gene [17]. We therefore deduced that the *GmCnx1* gene expression in soybean is probably related to the positive regulation on the resistance to SMV. In this study, SMV-1 and SMV-7 were used to test for plant resistance to SMV. Consistent with the results from the microarray experiments, our results showed that transgenic plants indeed have higher SMV resistance compared with non-transgenic plants after the virus infection (Fig 6). We suspect that resistance to SMV might be due to the increase in NR and AO activities which are enhanced by the overexpression of *GmCnx1* gene in soybean (Fig 7). In any case, this is the first report about the novel function of *GmCnx1* against SMV, which has apparent breeding implications in soybean.

Follow-up studies are required to elucidate the underlying mechanism of *GmCnx1* function in response to NR, AO activities and SMV resistance, and in particular, the exact role of AO activity in post-invasive cell wall defense to SMV. Here, we observed that *GmCnx1* gene enhanced SMV resistance of plants, but whether *Cnx2*, *Cnx3*, *Cnx6* and *Cnx7*, which function in the initial steps of Moco biosynthesis (Fig 7), had similar effects on SMV resistance remain unknown.

In conclusion, we show that overexpression of *GmCnx1* gene enhanced NR, AO activities and SMV resistance in soybean. This report provided valuable information on molecular breeding and improved our understanding of the biological and molecular functions of *GmCnx1*. These data provide a novel route for initiating molecular breeding to improve NR, AO activities and SMV resistance in soybean, a significant crop worldwide.

## Supporting Information

S1 Fig *Agrobacterium tumefaciens*-mediated transformation diagram using cotyledonary-node as explants in soybean. (A) Seed sterilization by chlorine gas method. (B) Seed germination on GM for 1 d. (C) *Agrobacterium tumefaciens* infection in LCCM for 30 min. (D) Co-cultivation explants with *Agrobacterium* for 4 d. (E) Multiple shoot induction on SI after two weeks. (F) Elongation of multiple shoot on SE after at least two weeks. (G) Elongation of multiple shoots. (H) Rooting on RM. (I) Plantlet domestication in greenhouse. (J) Plantlet transplanting and seed pod in greenhouse.
(TIFF)

S1 Table Agronomic performance of non-transgenic plants and *GmCnx1* transgenic plants. (XLSX)
