# Supplementary figures and images for: Overexpression of a GmCnx1 Gene Enhanced Activity of Nitrate Reductase and Aldehyde Oxidase, and Boosted Mosaic Virus Resistance in Soybean
Source: PLoS One. 2015 Apr 17;10(4):e0124273. doi: 10.1371/journal.pone.0124273 (PMC4401665; doi:10.1371/journal.pone.0124273)

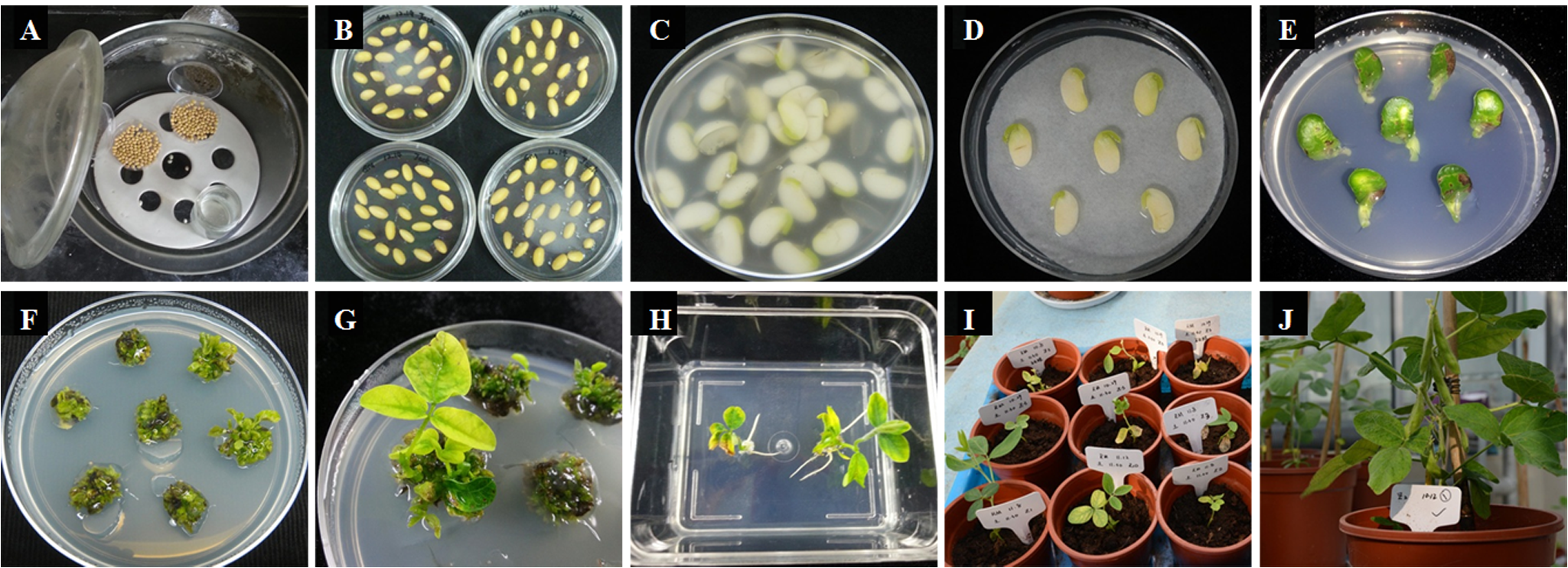

Supplement: S1 Fig — (A) Seed sterilization by chlorine gas method. (B) Seed germination on GM for 1 d. (C) Agrobacterium tumefaciens infection in LCCM for 30 min. (D) Co-cultivation explants with Agrobacterium for 4 d. (E) Multiple shoot induction on SI after two weeks. (F) Elongation of multiple shoot on SE after at least two weeks. (G) Elongation of multiple shoots. (H) Rooting on RM. (I) Plantlet domestication in greenhouse. (J) Plantlet transplanting and seed pod in greenhouse. (TIFF) [file pone.0124273.s001.tiff]
